# Layer-dependent stability of 2D mica nanosheets

**DOI:** 10.1038/s41598-023-34465-5

**Published:** 2023-05-15

**Authors:** Jae-Hun Kim, Vadym. V. Kulish, Shunnian Wu, Ping Wu, Yue Shi, Minoru Osada, Hyoun Woo Kim, Sang Sub Kim

**Affiliations:** 1 Department of Materials Science and Engineering, Inha University, Incheon, 22212 Republic of Korea; 2 Entropic Interface Group, Singapore University of Technology and Design, Singapore, 487372 Singapore; 3 Institute of Materials and Systems for Sustainability (IMaSS), Nagoya University, Nagoya, 464-8601 Japan; 4 Division of Materials Science and Engineering, Hanyang University, Seoul, 04763 Republic of Korea

**Keywords:** Materials science, Nanoscience and technology

## Abstract

We report on the layer-dependent stability of muscovite-type two-dimensional (2D) mica nanosheets (KAl_3_Si_3_O_10_(OH)_2_). First-principles calculations on mica nanosheets with different layer thicknesses (n = 1, 2, and 3) reveal their layer-dependent stability; odd-numbered 2D mica nanosheets are more stable than even-numbered ones, and the preferable stability of odd-numbered layers originates from electronic effects. A core-shielding model is proposed with a reasonable assumption, successfully proving the instability of the even-numbered mica nanosheets. Raman imaging supports that the population of odd-numbered mica nanosheets is predominant in exfoliated mica products. The alternating charge states with odd/even layers were evidenced by Kelvin probe force microscopy. We also demonstrate a unique photocatalytic degradation, opening new doors for environmental applications of mica nanosheets.

## Introduction

Atomic-layer control in two-dimensional (2D) nanosheets is receiving great attention as a new category of materials science because of the special physical and chemical properties of the nanosheets that suggest potential applications in next-generation electronic devices^1–11^. Triggered by the discovery of graphene^1,6^, many researchers have explored the properties and applications of 2D nanosheets, consisting of inorganic layered materials^4–8^. 2D materials have gained much consideration recently due to their extraordinary properties^11^ and they are used in different applications such as energy^12^, humidity sensor^13,14^, etc. Different characterization techniques such as optical methods^15^, Raman spectroscopy^16^, AFM study^17^, have been used to characterize them. Furthermore, their features are explored by density functional theory^18,19^.

In particular, being similar to graphene, it is expected that the reduction in the number of layers in the inorganic materials will bring about new properties and novel applications^20^. For instance, Ryousuke Ishikawa et al^21^ investigated the effect of the number of graphene layers on the performance of perovskite/graphene solar cells, in which they also report the layer dependent graphene workfunction; 4.82 eV for monolayer, 4.94 eV for bilayer, and a peak at approximately 4.97 eV for three and above layers. It is interesting to study if similar trends are kept in 2D inorganic structures.

Mica, owing to its peculiar properties including electrical, mechanical, and chemical stability, has been used in a variety of applications such as insulating substrates, capacitors, paint films, and barrier coatings. In particular, since mica is originally comprised of a layered structure with strong in-plane bonds and weak coupling between layers, it is efficiently cleaved into thin nanosheets^22–26^, presenting unique and useful properties. For example, with its dielectric characteristics with a bandgap of 7.85 eV^27–29^, the capacitive properties of mica will be extremely elevated by the exfoliation.

Even though there are some reports about the genuine exfoliation of mica nanosheets, down to single- or few-layered nanosheets^30,31^, there is still room for further studies in this aspect. Furthermore, there has been no significant progress toward the revelation of novel properties and applications of mica nanosheets with an extremely small thickness. In a recent publication^24^, we reported a simple but rather unexpected approach for tunable bandgap narrowing in muscovite-type (KAl_3_Si_3_O_10_(OH)_2_) 2D mica nanosheets through controlled molecular thickness. Decreasing the number of layers resulted in a reduced bandgap energy from 7 to 2.5 eV, and the bilayer case exhibited a semiconducting nature with a bandgap energy of ~ 2.5 eV. This was attributed to lattice relaxations, as well as surface-doping effects. These bandgap-engineered 2D mica nanosheets may solve some key issues in the development of 2D nanosheet-based electronic/optoelectronic devices that require a narrow bandgap, a semiconductor-to-metal transition, and excellent layer dependent structure stability.

In conjunction with that report, in this study, through first-principles density functional theory (DFT) calculations, we found that even-numbered 2D mica nanosheets are less stable than odd-numbered ones, which was explained by a core-shielding model and evidenced by Raman imaging.

## Materials and methods

### DFT calculations

First-principles calculations were performed within the DFT framework, as implemented in the the Vienna Ab-initio Simulation Package (VASP)^32^. The exchange–correlation potential was approximated by the generalized gradient approximation using the Perdew–Burke–Ernzerhof (PBE) functional^33–35^. The cut-off of the kinetic energy was set at 500 eV. All of the structures were treated with periodic boundary conditions, and mica nanosheets in the periodic supercells were separated by a vacuum spacing of being about 20 Å. The crystal lattice parameters of 1L, 2L and 3L mica nanosheets are available in our previous report^36^. The optimized structures were obtained by relaxing all atomic positions until the interatomic forces became less than 0.01 eV Å^−1^. The K-point meshes for geometric structure optimization and electronic structure calculations are 6 × 4 × 1 and 10 × 6 × 1 respectively. The charge in an atom was defined as the difference between the valence charge and the Bader charge. The Bader charge was determined with the Bader scheme of charge density decomposition^37^.

### Chemical exfoliation

The chemical exfoliation process of the mica nanosheets is described in detail elsewhere^2^. In a Teflon-lined autoclave with a mixture of potassium organic solution and mica powders, the dissolved potassium ions in the solvent were intercalated into the interlayer space of the mica. Simultaneously, the mica was irradiated by a rapid microwave heating, generating the exfoliated mica nanosheets. A colloidal suspension of thin mica layers was then acquired by centrifugation and sonication.

### Mechanical exfoliation

We obtained the ultrathin mica nanosheets by means of the mechanical exfoliation of muscovite mica with Scotch tape. As a source material, commercially available, highly ordered mica plates were used.

### Characterization

Optical microscopy (Olympus BX-51) and AFM (SII Nanotechnology E-sweep AFM) were used to observe the topography of the nanosheets. Raman spectra were collected by using a HORIBA Jobin Yvon (LabRam HR800UV) Raman microscope with an excitation wavelength of 514.5 nm. Kelvin probe force microscopy (KPFM) was carried out with an SII Nanotechnology E-sweep AFM in vacuum to avoid contamination and water adsorption.

### Photocatalytic experiments

The raw materials are as follows; TiO_2_ (Degussa P-25) as a reference material, exfoliated mica layers, and methylene red (MR) solution that was purchased from Daejung Chemicals & Metals Co., Ltd., Korea. According to the specifications, the MR solution consisted of 90% ethanol, 9.9% deionized water, and 0.1% MR. In a 250-mL conical flask, 6 mg of photocatalytic powder (TiO_2_ (Degussa P-25) or exfoliated mica layers) was suspended in 50 mL of the MR solution kept in darkness at room temperature with stirring for 1 h. Then, the mixtures were kept under sunlight between 11.00 and 15.00 pm at the campus of Inha University, Republic of Korea; the experiments using MR were carried out on different days. The aliquots from the aqueous solution were taken out at intervals (30, 60, 120, 180, and 240 min), filtered with a filter paper, and the UV–vis absorption spectrum of the clear solution with the same amount was recorded by a UV–vis spectrometer (VARIAM Technology, Cary 100 UV–vis). The photocatalytic activity of the synthesized photocatalyst was determined by the decrease in the absorbance at 518 nm for MR.

## Results and discussion

### First-principles calculations and modeling of 2D mica nanosheets

In an earlier study^24^, we carried out first-principles DFT calculations on the structural and electronic properties of muscovite-type (KAl_3_Si_3_O_10_(OH)_2_) 2D mica nanosheets with a controlled molecular thickness. In the 2D mica nanosheets with different numbers of layers, an abnormal bandgap narrowing was observed, contrary to well-known quantum size effects. Decreasing the number of layers resulted in a reduced bandgap energy from 7 to 2.5 eV, proposing a novel approach to preparing 2D materials with smaller bandgaps, which are needed to prepare devices from 2D heterostructures.

Since mica nanosheets are exfoliated from the host crystallites, they may present different structural properties due to the fact that a large proportion of the atoms are now situated on the surface, as opposed to being confined within the bulk. Accordingly, the structural distortions in the mica nanosheets may contribute to their counterintuitive band narrowing observed in an earlier study^24^ and change their stability as well. In order to investigate and compare the relative stabilities of single- and few-layered mica nanosheets, we performed a series of calculations on single-layered (1L), double-layered (2L), and triple-layered (3L) mica nanosheets as model systems. We calculated the surface energy of mica nanosheets to quantify the stability by using the following equation:

1 $$\Delta {H}_{s}=\frac{1}{2A}\left({E}_{slab}-N{E}_{bulk}\right)$$

where A is the area of the slab supercell surface, E_slab_ is the energy of the slab supercell, E_bulk_ is the bulk energy per atom, and N is the number of atoms in the slab supercell. Interestingly, we found that 2L mica is 36 and 22 meV less stable than 1L and 3L mica, respectively. The stability characterized by the surface energy can be assumed to be derived from two factors, structural and electronic:

2 $$\Delta {H}_{s}\propto {E}_{structural}+{E}_{electronic}$$

where the E_structural_ component originates from the local atomic environment at the surfaces (i.e., from the presence of dangling bonds, chemical potentials, etc.), while E_electronic_ comes from quantum electronic effects (i.e., quantum confinement, etc.). In our case, all single-, double-, and triple-layered mica structures have the same kind of K^+^ termination; hence, we can rule out the E_structural_ factor. Therefore, the preferable stability of odd-number-layered mica sheets should originate from the electronic effects. To support this hypothesis, we have developed a core-shielding model for the description of few-layered mica. According to this model, we treat the mica structure as a sequence of charged positive and negative layers. The positive layer corresponds to the K^+^ region, while the negative layer corresponds to a tetrahedral–octahedral–tetrahedral sandwich region (Fig. 1 in Ref.^24^). The positive and negative regions interact with each other according to a general Coulomb-like equation:

3 $$\sigma {E}_{1}\propto -\frac{{Q}_{1}{Q}_{2}}{r}$$

Therefore, the stability depends on the values of positive and negative charges and the distance between the positive and negative layers. In single-layered mica nanosheets (1L), there is a stable electromagnetic attraction between the positive surface K^+^ ion layer (Q_1_) and the negative tetrahedral–octahedral–tetrahedral sandwich core (Q_2_) charges. However, in double-layered mica nanosheets (2L), there is a positive charge in the core region. Although it is screened by the neighbouring negative charges, this shielded positive charge in the core repels the positive K^+^ ion layer in the surface, making double-layered mica unstable. The shielding effect is equivalent to partial deduction of the negative Q_2_, therefore, the equation above can be re-written as follows:

4 $$\sigma {E}_{2}\propto -\frac{{Q}_{1}\left({Q}_{2}-{Q}_{2}^{shielded}\right)}{r}$$

This brings about a decrease of the attractive electrostatic interaction between the surface K^+^ ion layer and the neighbouring negative tetrahedral–octahedral–tetrahedral sandwich layer. Further, in triple-layered mica nanosheets (3L), a negative charge occurs in the core region. It is definitely screened by the neighbouring positive K^+^ ion layer before this shielded negative charge in the core attracts the positive K^+^ ion layer in the surface. However, the far distance may dramatically weaken the attractive interaction to stabilize the 3L structure.

**Figure 1:**
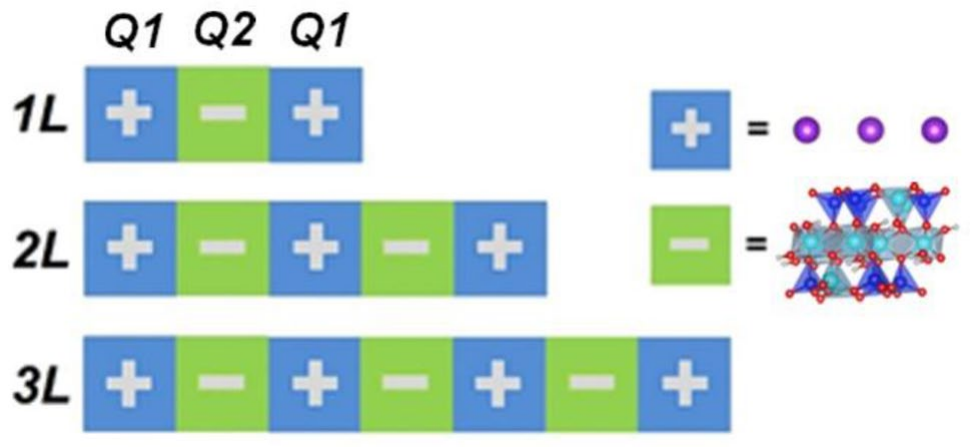
Core-shielding model for few-layered mica. The blue color represents positive-charge regions (K layer) and the green color represents negative-charge regions (tetrahedral and octahedral sandwich structure).

To verify this hypothesis, we calculated the Bader charges on all atoms in single-, double-, and triple-layered mica. A negative value of Bader charge stands for acceptance of electrons to yield  charge, while a positive value represents donation of electrons to give  charge. We found that the surface K atoms in all cases possess a similar charge of + 0.89|e|, indicating that they are almost completely ionized. This also suggests equal Q1 values of Eq. 1 in all cases. The charge of the core region in 1L has a value Q_2_ of being − 1.78|e|, giving the attractive electrostatic interaction between the K^+^ ions and the core. However, a ${Q}_{2}^{shielded}$ value of 0.004|e| in 2L, the indication of the shielding effect of negative layers impacting on the core positive charge, leads to a reduction of Q_2_. As a result, we expect a much weaker interaction obtained in 2L with Eq. (4), hence, less stability. The calculated ${Q}_{2}^{shielded}$ value in 3L is negligible, which can be attributed to the large distance between the core to the surface. This implicates that the core-shielding effect may not be substantial in mica nanosheets of many layers. On this basis, we may expand our conclusion further and suggest that mica with an even number of layers would be less stable than mica with an odd number of layers (Fig. 1).

### Population of even- or odd-numbered mica nanosheets

**Figure 2:**
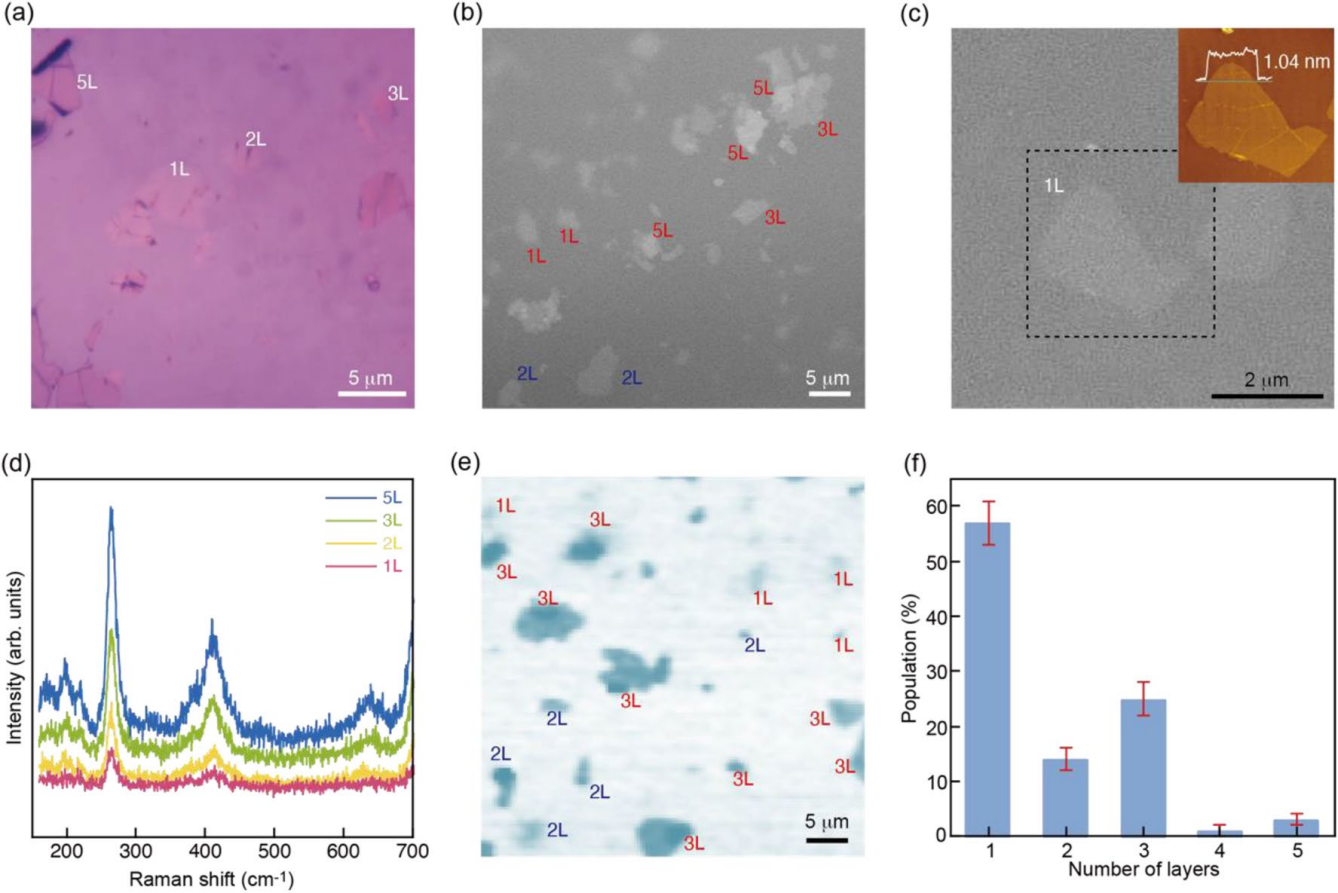
(**a**) Optical image measured with a white light on mica nanosheets on a 100 nm SiO_2_/Si substrate. (**b**) Optical image measured with the 530 nm light, which is near the maximal contrast for mica nanosheets. (**c**) Optical image measured with the 530 nm light on monolayer mica nanosheets. (inset) AFM image taken from the dotted area in (c). (**d**) Raman spectra of mica nanosheets with different layer numbers. The spectra were taken from the same film as (b). (**e**) Raman intensity maps monitored with 263-cm^−1^ mode. (**f**) Distribution of the layer numbers estimated from 100 Raman mapping images.

For the purpose of proving the theoretical calculation that predicts that odd-numbered mica layers are more stable than even-numbered ones, we have attempted to measure the population of each layer in the product of exfoliated mica nanosheets. In order to identify the number of layers in the exfoliated mica product, we performed a combined characterization of optical reflectivity and Raman scattering. Figure 2a shows an optical image under a while light illumination. It was observed that the apparent color depends on nanosheet thickness, due to the interference effects from mica/SiO_2_ and SiO_2_/Si interfaces^25,26^. To get more quantitative picture of the thickness distribution, we performed optical characterizations (Fig. 2b,c) using a monochromatic illumination at 530 nm, which is near the maximal contrast for mica nanosheets^25^. Clearly, mica nanosheets showed higher reflectivity than the substrate. This image provides a clear picture of thickness distribution with layer ranging from 1 to 5L. The inset of Fig. 2c shows an AFM image measured from 1L part of the optical contrast. The height different between mica nanosheet and SiO_2_/Si substrate was 1.04 nm, which was comparable with the theoretical thickness of the 1L mica nanosheet^24^.

Having established the thickness identification using the optical reflectivity, we extend Raman spectroscopy, which is one of the powerful tools for the thickness characterization of 2D materials. Figure 2d shows Raman spectra of mica nanosheets with different layer numbers. Although Raman scattering efficiency of mica is much lower (approximately 100 times) than those of graphene and MoS_2_, we could monitor the thickness dependence; the peak intensity in the Raman spectra is evidently dependent on the number of layers. By converting the peak intensity into an image, one can visualize the population of the mica nanosheets in terms of the number of layers, which is often used in determining the number of layers in 2D materials. On this basis, we can make a Raman imaging, indicating the number of layers for exfoliated mica products. One example of the Raman imaging is shown in Fig. 2e, revealing the existence of few-layered mica nanosheets. The typical numbers of mica nanosheets were observed to be 1, 2, 3, and 5. We observe that mica nanosheets with an even number of layers (i.e., 2 in the present case) have been produced, in addition to those with an odd number of layers (i.e., 1, 3, and 5). Although the results confirm the existence of 2L mica nanosheets, the amount of odd-layered mica nanosheets is significantly larger than that of even-layered ones. We have estimated the population of odd and even layers from 100 Raman images, which were taken from different exfoliated mica products. The results are summarized in Fig. 2f, evidently demonstrating that the odd-numbered mica nanosheets are predominant. This direct observation of the population of mica nanosheets in terms of the number of layers is well consistent with the core-shielding model, indicating that even-layered mica nanosheets are less stable in comparison to the odd-layered ones.

### Surface charge states of mica nanosheets

**Figure 3:**
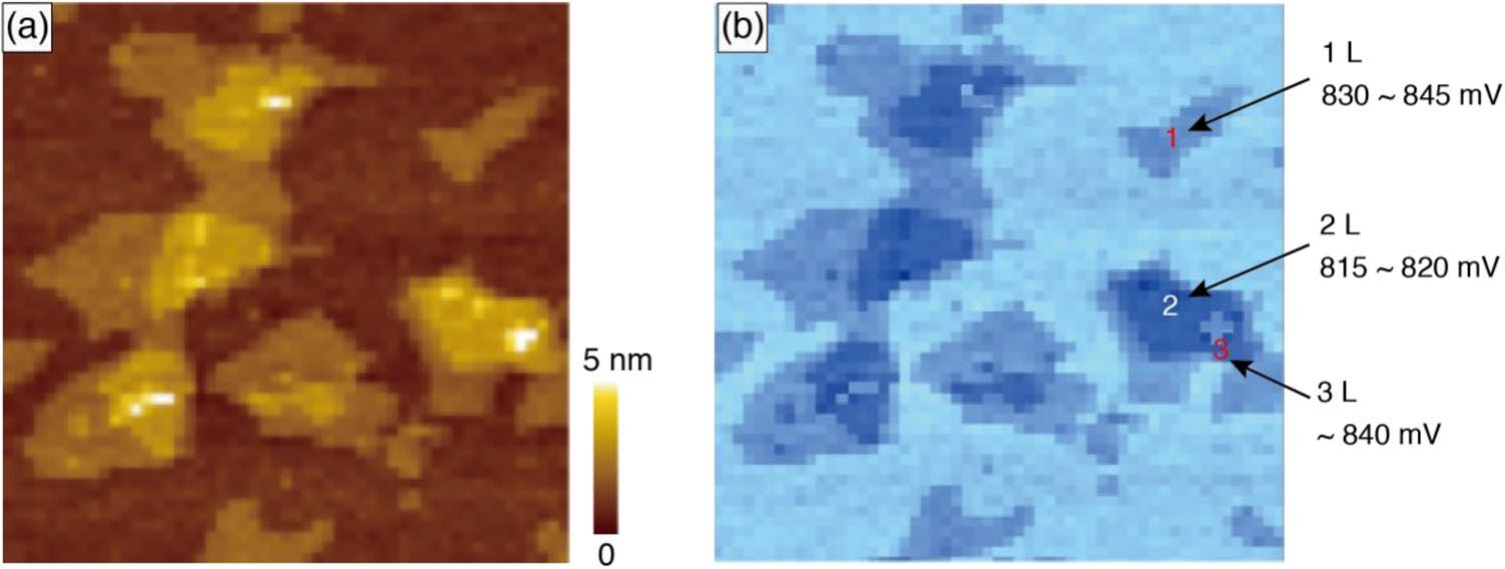
(**a**) AFM image and (**b**) charge state measured by KPFM of the exfoliated mica product.

According to the core-shielding model, due to the alternation of charges in the core region that may lead to the alternation in dipole fields with components parallel to the surface, there may be two-type surface charges for odd/even numbered layers. That is, the surfaces of all types of layers are all positive, but the odd-numbered layers have a strong dipole due to the more negative core regions, and the even-numbered layers have a relatively weak dipole due to the less negative core regions. To verify this, we have measured the surface charge state of the 2D mica nanosheets by using Kelvin probe force microscopy (KPFM), with which one can observe the work function of surfaces at a molecular scale. The map of the work function produced by KPFM gives us information about the composition and electronic state of the local structures on the surface of a solid because the work function of a solid is determined by various surface phenomena, chemical composition, reconstruction of surfaces, and doping and band-bending of semiconductors. As is shown in Fig. 3, odd-numbered layers such as 1L and 3L mica nanosheets are more positive than even-numbered layers such as 2L mica nanosheets. What is more important is that the surface charge states for 1L and 3L are almost identical. We proved that the 3L nanosheet has a larger bandgap than the 1L and 2L ones; if the surface charge does not play a role in Fig. 3, we should see 3 types (one for each layer type) corresponding to the 3 work functions. The fact that there are two types observed in Fig. 3 is in favor of the alternating charges with the odd/even layers.

### Dye degradation

**Figure 4:**
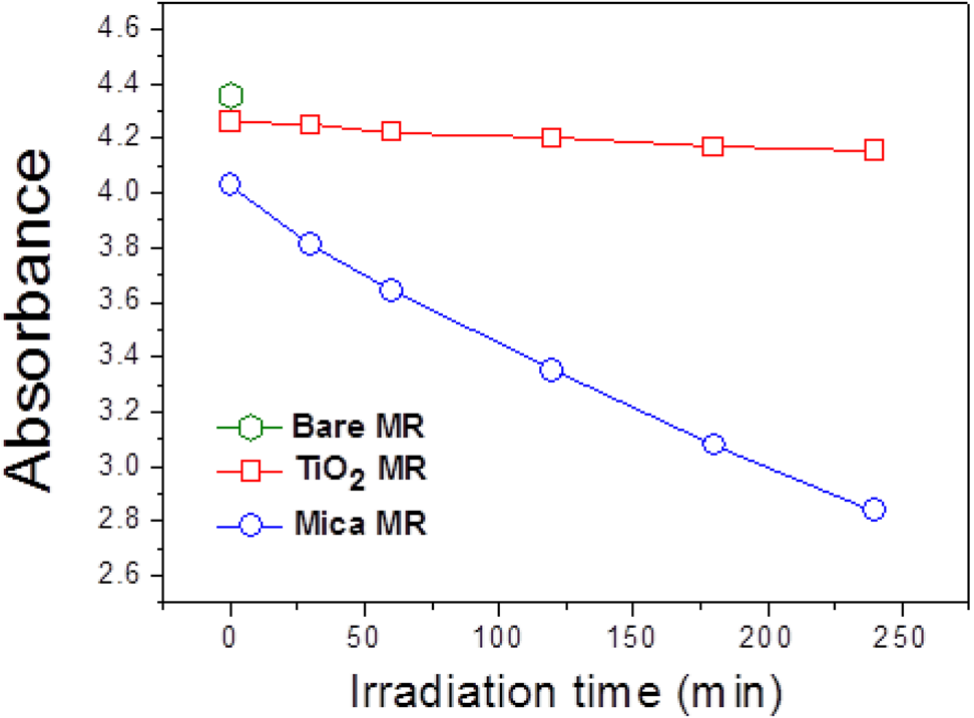
UV–vis absorption spectra of MR at 518 nm.

For the purpose of exploiting the exfoliated mica nanosheets, we have investigated their photocatalytic properties by testing dye degradation. The photocatalytic degradation activities of the mica and TiO_2_ (used as a reference) are shown in Fig. 4. The ultraviolet–visible (UV–vis) absorption spectra of methylene red (MR) solutions containing TiO_2_ or mica nanosheets are shown in Fig. S1 (see Supplementary Fig. S1 online). Prior to the sunlight irradiation, the suspensions of mica or TiO_2_ and the dye-containing MR solution were stirred in the dark to establish the adsorption/desorption equilibrium between the dye and the photocatalyst.

As shown in Fig. 4, mica nanosheets demonstrate a strong absorption of the anionic MR dye with the initial absorption value of 4.05. Particularly, the absorbance of MR on the mica nanosheets decreased from 4.05 to 2.8 after 250 min of sunlight irradiation. On the other hand, MR did not degrade at all on TiO_2_ and its concentration remained nearly unchanged after 250 min. Dye degradation needs two steps: (1) dye adsorption on the mica nanosheet and (2) dye destruction by photoelectrons. The mica nanosheets prepared by exfoliation consist of few-layered flakes, being composed of mainly 1 and 3 layers and partly of 2 layers. According to our *“core-shielding”* model, the mica nanosheet structure can be treated as a sequence of charged positive and negative layers. The positive layer corresponds to the K^+^ region, while the negative layer corresponds to a sandwiched SiO_4_–AlO_6_–SiO_4_ region. Accordingly, the effective MR degradation by the exfoliated mica nanosheets is reasonable. In contrast, we do not expect significant degradation of MR on the negatively-charged TiO_2_ surface. Our studies on mica nanosheets suggest it to be a potential candidate that can effectively degrade acidic dyes. Since a positive charged TiO_2_ surface would yield a better understanding of the positive mica surface effects to photocatalytic degradation of anionic MR dye, we plan to synthesize positive charged TiO_2_ nanoparticles by adjusting the pH of solution^38^, or incorporating protonated amine^39^ for their photocatalytic dye degradation tests.

Since the surface K atoms in 1L, 2L and 3L mica nanosheets possess a similar charge of + 0.89|e|, their corresponding electrostatic interaction to bring about surface adsorption of anionic MO dye may show insignificant discrepancy. Considering that the bandgap energy tends to reduce with the decrease of layer numbers^24^, this will lead to an increase of UV–Vis light adsorption. Therefore, assuming that the electron–hole pair recombination would not alter with the number of nanosheets layers, the efficiency of photocatalytic degradation of MO dye will increase with the decrease of the number of layers.

## Conclusions

We investigated the layer-dependent stability of muscovite-type (KAl_3_Si_3_O_10_(OH)_2_) 2D mica nanosheets using DFT-based theoretical modeling and spectroscopy techniques. DFT calculations on mica nanosheets with different layer thicknesses (n = 1, 2, and 3) indicated that the preferable stability of odd-numbered layers originates from electronic effects. With a reasonable assumption that the electronic effects will play a major role, we developed a core-shielding model, successfully proving the instability of the even-numbered layers of mica nanosheets; the K interlayer inside double-layered mica creates a shielding effect for the core charge. Raman imaging revealed that the exfoliated mica products are comprised predominantly of odd-numbered layers (i.e., 1L, 3L, and 5L), as well as a minor amount of even-numbered ones (such as 2L and 4L), being consistent with the model, which was further evidenced by the surface charge measurement via KPFM. We have compared the photocatalytic degradation ability of exfoliated mica nanosheets to that of TiO_2_ by using a MR solution. Mica had a very high degradation ability toward MR, whereas TiO_2_ did not, indicating that the mica nanosheets have a potential for use in the photocatalytic degradation of acidic MR.

## Supplementary Information

Supplementary Information.

## Data Availability

All data generated or analyzed during this study are available from the corresponding author upon reasonable request.
