# Supplementary material for: Layer-dependent stability of 2D mica nanosheets
Source: Sci Rep. 2023 May 15;13:7880. doi: 10.1038/s41598-023-34465-5 (PMC10185575; doi:10.1038/s41598-023-34465-5)
Supplement: Supplementary file 1 — Supplementary Information. [file 41598_2023_34465_MOESM1_ESM.docx]

Supplementary materials

Layer-dependent stability of 2D mica nanosheets

Jae-Hun Kim^1^, Vadym. V. Kulish^2^, Shunnian Wu^2^, Ping Wu^2,^*, Yue Shi^3^, Minoru Osada^3,*^, Hyoun Woo Kim^4,*^, Sang Sub Kim^1,^*

*^1^Department of Materials Science and Engineering, Inha University, Incheon 22212, Republic of Korea.*

*^2^Entropic Interface Group, Singapore University of Technology & Design, Singapore 487372, Singapore.*

*^3^Institute of Materials and Systems for Sustainability (IMaSS), Nagoya University, Nagoya 464-8601, Japan.*

*^4^Division of Materials Science and Engineering, Hanyang University, Seoul 04763, Republic of Korea.*

*Correspondence and requests for materials should be addressed to Ping Wu (wuping@sutd.edu.sg), Minoru Osada (mosada@imass.nagoya-u.ac.jp), Hyoun Woo Kim (hyounwoo@hanyang.ac.kr), or Sang Sub Kim (sangsub@inha.ac.kr),*


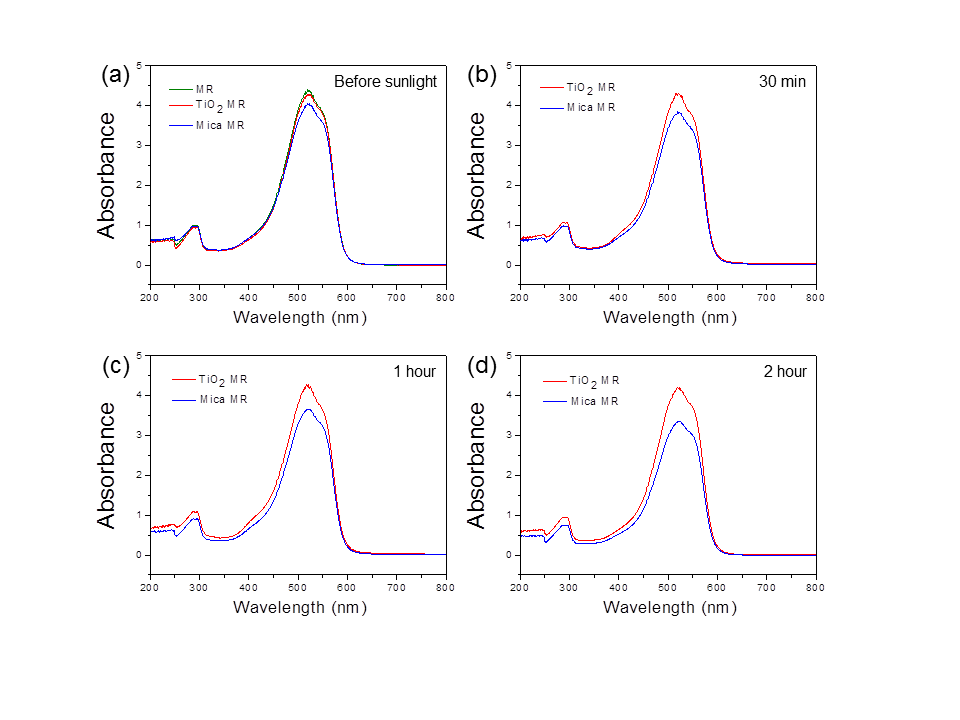


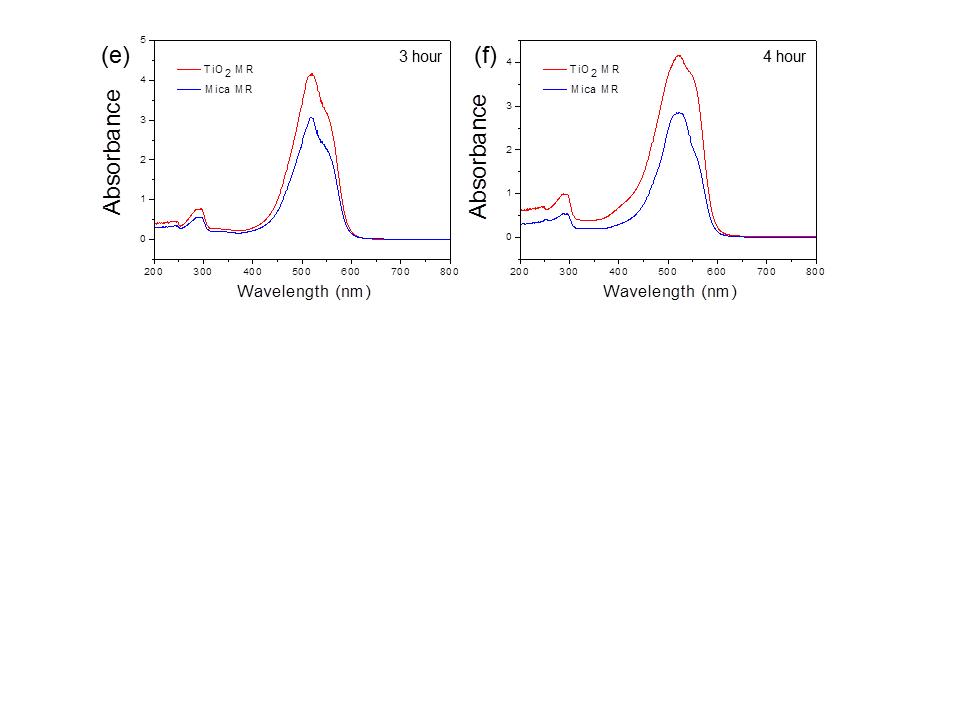


**Figure S1.** UV–vis absorption spectra of MR solution containing TiO_2_ or mica nanosheets.
